# Cancer Cachexia and Related Metabolic Dysfunction

**DOI:** 10.3390/ijms21072321

**Published:** 2020-03-27

**Authors:** Guilherme Wesley Peixoto da Fonseca, Jerneja Farkas, Eva Dora, Stephan von Haehling, Mitja Lainscak

**Affiliations:** 1 Heart Institute (InCor), University of São Paulo Medical School, São Paulo SP 05403-900, Brazil or; 2 Department of Cardiology and Pneumology, University Medicine Göttingen (UMG), DE-37075 Goettingen, Germany; 3 Research Unit, General Hospital Murska Sobota, SI-9000 Murska Sobota, Slovenia; jerneja.farkas@sb-ms.si; 4 National Institute of Public Health, SI-1000 Ljubljana, Slovenia; 5 Faculty of Medicine, University of Ljubljana, SI-1000 Ljubljana, Slovenia; 6 Division of Cardiology, General Hospital Murska Sobota, SI-9000 Murska Sobota, Slovenia; dora.evaa@gmail.com; 7 German Center for Cardiovascular Research (DZHK), partner site Goettingen, DE-37099 Goettingen, Germany

**Keywords:** cancer cachexia, metabolic dysfunction, inflammation, metabolism, clinical management

## Abstract

Cancer cachexia is a complex multifactorial syndrome marked by a continuous depletion of skeletal muscle mass associated, in some cases, with a reduction in fat mass. It is irreversible by nutritional support alone and affects up to 74% of patients with cancer—dependent on the underlying type of cancer—and is associated with physical function impairment, reduced response to cancer-related therapy, and higher mortality. Organs, like muscle, adipose tissue, and liver, play an important role in the progression of cancer cachexia by exacerbating the pro- and anti-inflammatory response initially activated by the tumor and the immune system of the host. Moreover, this metabolic dysfunction is produced by alterations in glucose, lipids, and protein metabolism that, when maintained chronically, may lead to the loss of skeletal muscle and adipose tissue. Although a couple of drugs have yielded positive results in increasing lean body mass with limited impact on physical function, a single therapy has not lead to effective treatment of this condition. Therefore, a multimodal intervention, including pharmacological agents, nutritional support, and physical exercise, may be a reasonable approach for future studies to better understand and prevent the wasting of body compartments in patients with cancer cachexia.

## 1. Introduction

Cancer cachexia is a complex multifactorial syndrome that leads to substantial and unintentional body weight loss, marked by a continuous depletion of skeletal muscle mass associated in many, but not all, cases with a reduction in fat mass [1]. This loss of body weight is irreversible by nutritional support alone and leads to progressive functional impairment. Cancer cachexia is estimated to affect up to 74% of patients with many types of cancer globally, with the highest incidence in head and neck, pancreatic, gastric, and hepatic cancer [2]. Moreover, cancer cachexia is associated with impaired physical function, increased risk of treatment-related complications, as well as higher rates in hospitalizations and mortality [3,4,5,6].

To date, there is no final agreement regarding the definition of cancer cachexia, however, commonly used criteria to define cachexia are (1) patients who have lost more than 5% of body weight over the last 6 months or (2) presence of either body mass index (BMI) lower than 20 kg/m² or sarcopenia associated with ongoing weight loss of more than 2% [1]. Despite validation of BMI and weight loss as criteria to distinguish cachectic and non-cachectic patients [7], a recent study in patients with pancreatic cancer challenges the aforementioned criteria; with computed tomography analysis, a tissue loss of more than 5% was detected in 81% of patients while the traditional definition identified only 57% of patients as being cachexic [8]. It seems that more precise assessment of body composition should be applied along the tissue wasting trajectory in patients with cancer to detect cachexia as early as possible [9].

Although the molecular mechanisms involved in the development and progression of cancer cachexia have not been elucidated in detail, it is suggested that the interaction between cancer cells and other organs, especially muscle and fat tissue, promotes alterations in body composition seen in these patients [10]. Skeletal muscle counted as a whole is the largest organ of the human body and plays a critical role in controlling metabolism in patients with cancer cachexia. Additionally, tumor cells can switch energy production from oxidative phosphorylation to cytosolic glycolysis, forcing the organism to heavily depend on glucose as its main source of fuel [11]. Thus, this metabolic derangement mobilizes glucose precursors from muscle and adipose tissue that may lead to loss of body weight when sustained chronically. Moreover, this metabolic responses seem to be mediated by secretion of pro-inflammatory cytokines from cancer cells and also from the immune system of the host, including tumor necrosis factor (TNF), interferon-gamma (IFN-γ), and several interleukins (IL-6, IL-1β) [12]. Activin and myostatin, other catabolic factors driven by the tumor, have also been described as mediators of metabolic derangement [13].

The aim of this review is to outline the metabolic disturbances commonly reported in patients with cancer cachexia and to elucidate factors that may contribute to cancer-related metabolic dysfunction with tissue loss within body compartments. 

## 2. Altered Energy Balance

Overall, patients with cancer present considerable changes in the homeostasis of energy production and consumption, favoring a negative energy balance [14]. The total daily energy expenditure is composed of three components: (1) resting energy expenditure, (2) energy expenditure during physical activities, and (3) the thermogenic effect of food. In addition, the chronically increased energy imbalance, often described in patients with cancer cachexia [15], is attributed to either a decrease in energy intake or an elevated resting energy expenditure due to tumor metabolism, and the combination of both can also occur [16,17].

The energy demand of a tumor may influence energy expenditure and initiate processes of body wasting. Through proteolysis, the muscle is degraded into amino acids that serve as a fuel through hepatic gluconeogenesis. In parallel with proteolysis, there is a breakdown of triacylglycerol (lipolysis) into three molecules of free fatty acids and one of glycerol, the free fatty acid molecules are later oxidized and glycerol is used as an energy source for gluconeogenesis as well. Additionally, increased muscle glycolysis even in the presence of oxygen, the so-called Warburg effect, leads to higher production of lactic acid, which is further converted into glucose via the Cori cycle in the liver [18]. In addition, mitochondrial dysfunction may also increase the production of lactic acid by reduced efficiency in extracting energy via the Krebs cycle [19]. Therefore, tumors can increase the global rate of glycolysis, glycogenolysis, lipolysis, and proteolysis with the purpose of recycling glucose via gluconeogenesis [20].

These metabolic alterations, however, are distinguished from episodes of starvation/fasting or caloric restriction, where fat storage replaces glucose as the primary source of fuel after glycogen depletion, followed by significant protein breakdown in more advanced phases during which also resting energy expenditure is adjusted to accompany the deficiency in energy supply [21,22]. On the contrary, patients with cancer cachexia present increased lipolysis with increased mRNA expression of the hormone-sensitive lipase enzyme (HSL) and elevation in β oxidation of free fatty acids [23,24]. Affected patients frequently have increased proteolysis associated with reduced protein synthesis [25]. Thus, in this cascade of metabolic events, cancer cachexia has been quoted as a state of “autocannibalism” in which catabolic metabolism takes place to improve anabolic tumor metabolism [12].

Recently, a hybrid metabolic state, defined by the capability of cancer cells to switch between oxidative and glycolytic metabolism, has been proposed to explain how cancer cells can adapt to distinct microenvironments [26]. Even though cells under physiological conditions tend to be more oxidative or glycolytic depending on their metabolic function and substrate availability, cancer cells may have a hybrid state, allowing them to develop a phenotype with both metabolic features to support survival and proliferation [27]. Moreover, cancer cells seem to upregulate plasma membrane transporters of glucose, lactate, and amino acid with the purpose to support their growth [28].

It is important to acknowledge that the energetic cost of a tumor for the host may range from 100–1400 kcal/day [29]. The tumor type also plays a role in altering energy balance, once a higher resting energy expenditure has been demonstrated in patients with lung cancer when compared to patients with gastrointestinal cancer [30]. In addition, central tumor localization, compared to peripheral localization, in patients with lung cancer also showed a more pronounced increase in energy expenditure [16]. Although we can assume that energy imbalance may be more frequent in certain types of cancer (i.e., lung cancer), further studies assessing energy metabolism in cancer subpopulations are necessary. In fact, a hypermetabolic state is a common feature in patients with cancer, although a lower percentage of patients may develop hypometabolism or no changes at all [31].

The Harris-Benedict equation has been largely used to estimate the energy expenditure in patients with cancer in spite of its limitations to predict energy expenditure in malnourished patients [32]. A predicted resting energy expenditure higher than 110% has been described in 58% of patients with unselected cancer [33] and it has been independently associated with poor prognosis in a recent study with metastatic non-small cell lung cancer (NSCLC) [34]. However, a more appropriated analysis would be to correct measured resting energy expenditure (by indirect calorimetry) for lean body mass (LBM), considering that patients with cancer might present a daily energy consumption of 43.7 kcal/kg [34].

Skeletal muscle mass is the largest contributor to resting energy expenditure and along with adipose tissue an energetic reservoir for many bodily functions. A study in patients with stage III and IV of head and neck cancer assessed the reduction in LBM and fat mass after one month of concurrent chemo-radiation treatment and found a reduction by 71.7% and 28.3%, respectively [35]. In addition, muscle wasting is not an exclusive feature of skeletal muscle involved in locomotion, it has also been shown to affect respiratory muscles and even myocardium in patients with chronic disease-associated wasting [36,37,38]. Additionally, the progression of tumor cells, as well as cardiotoxicity induced by cancer treatment, may lead to a condition called “cardiac cachexia” that is characterized by cardiac atrophy, fibrosis, and myocardial dysfunction [39].

**Figure 1:**
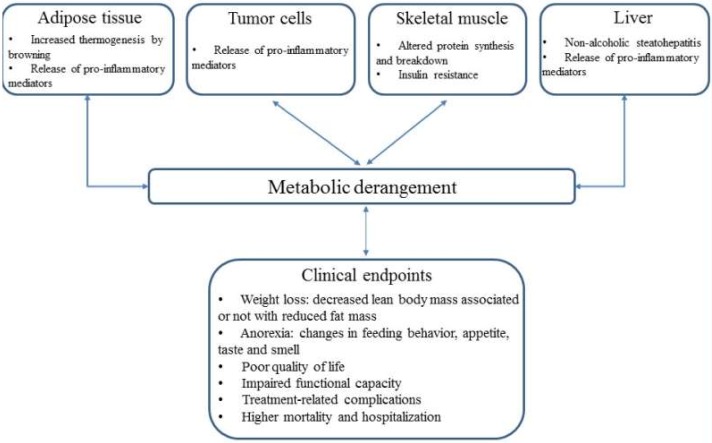
Metabolic derangement as a result of byproducts released from adipose tissue, tumor cells, skeletal muscle and liver, leading to clinical endpoints.

In light of these findings, the metabolic derangement seems to lead to a decline in global skeletal muscle mass followed by loss of adipose tissue to a lesser degree (Figure 1), though other studies have shown that the loss of fat mass can occur without reductions in muscle mass in 17% of patients with pancreatic cancer [8]. However, the order and contribution of each body compartment in the course of wasting still need to be determined by further studies.

Furthermore, cancer cachexia is often associated with anorexia and, in many cases, it is not only a matter of inadequate food intake [40]. Anorexia has shown to impact appetite, taste, and smell in these patients [41]. The mechanism for these modifications seems to operate on a central level, once pro-inflammatory factors released by the tumor may cross the blood–brain barrier and act on the hypothalamus through pro-opiomelanocortin neurons, reducing appetite and feeding behavior [42,43].

On the other hand, the typical skinny patient with wasting-related complications associated with cancer cachexia may not always be the case. Indeed, cachectic patients can be obese, causing an elusive “protection” due to a phenomenon known as the obesity paradox [9]. In a population of patients with pancreatic cancer, 16.2% of patients developed sarcopenia associated with obesity and the presence of sarcopenic obesity has also been shown to be an independent indicator of adverse prognosis in this population [44].

## 3. Inflammatory Markers and Muscle Mass

Under healthy physiological conditions, macrophages and dendritic cells, part of the innate immune system, are responsible for detecting agents that might cause infection and tissue damage in the organism. As a result of this, they produce an immune response via pro-inflammatory cytokines release, such as IL-6, IL-1β, TNF, and IFN-γ, and these inflammatory markers might act in nearby (paracrine action) or distant tissues (endocrine action). Likewise, the tumor microenvironment releases cytokines that contribute to their growth and favor the energy supply for them [45]. Based on this context, this section aims to describe the inflammatory pathways within skeletal muscle that may lead to muscle wasting.

**Figure 2:**
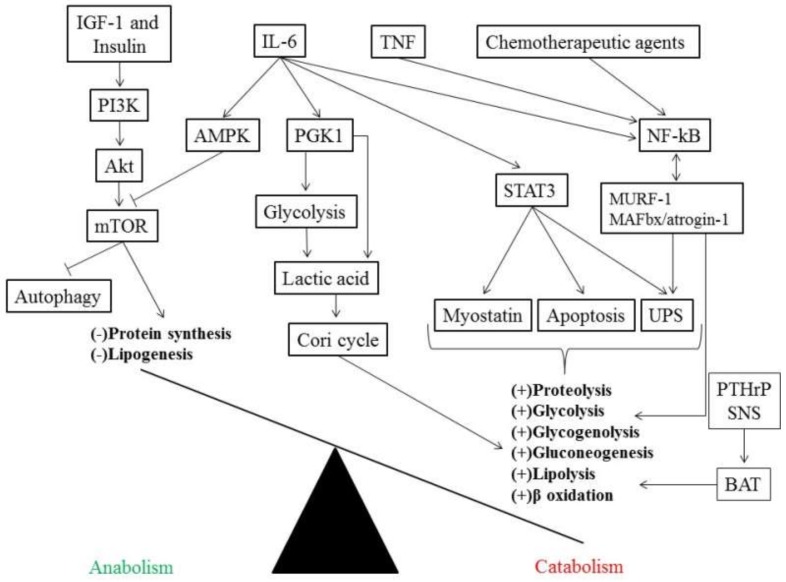
Pro-inflammatory mediators causing an energetic imbalance between catabolic (in the bottom right side in bold) and anabolic (in the bottom left side in bold) pathways. Akt, Protein kinase B; AMPK, AMP-activated protein kinase; BAT, brown adipose tissue; IGF-1, insulin-like growth factor 1; IL-6, interleukin-6; MAFbx, muscle atrophy F-box; mTOR, mammalian target of rapamycin; MuRF-1, muscle RING Finger-1; NF-κB, nuclear factor kappa-light-chain-enhancer of activated B cells; PGK 1, phosphoglycerate kinase 1; PI3K, phosphoinositide 3-kinase; PTHrP, tumor-derived parathyroid-hormone-related protein; SNS, sympathetic nervous system; STAT3, activating the signal transducer and activator of transcription 3; TNF, tumor necrosis factor; UPS, ubiquitin-proteasome system.

In patients with cancer, IL-6 binds to its receptor IL-6R and exerts its effect by activating the signal transducer and activator of transcription 3 (STAT3) (Figure 2) [46]. STAT3 interacts with its receptor (glycoprotein 130) through janus kinases (JAKs) phosphorylating the specific tyrosine residue (Tyr 705), and as a result, triggers the transcription of specific genes related to cell proliferation, cell growth and inhibition of apoptosis [47]. Moreover, other mediators can also activate STAT3 such as IL-2, IL-10, epidermal growth factor (EGF), and IFN-γ [48]. Interestingly, STAT3 can increase, in a positive feedback fashion, the expression of genes involved in its own activation, including IL-6, IL-10, and EGF [49].

Although the development and progression of cancer cachexia due to overactivation of STAT3 is unclear, increased activation of STAT3 has been associated with loss of muscle mass in several mice models [50,51,52]. In addition, STAT3 has been shown to be associated with two important proteolysis pathways, including the ubiquitin-proteasome system (UPS) and apoptosis through activation of caspase-3 (Figure 2), whereas blocking STAT3 preserved muscle mass in a mouse model of C26 colon carcinoma and Lewis lung carcinoma cells [50]. Moreover, STAT3 has also been described to be related to loss of muscle mass by stimulating myostatin, a strong inhibitor of myogenesis [53], while moderate-intensity exercise in mice was able to maintain muscle mass in spite of increased STAT3 activity [54].

IL-6 secretion has also been shown to directly regulate energy homeostasis by phosphorylating phosphoglycerate kinase 1 (PGK 1), which is an enzyme responsible for generating adenosine triphosphate (ATP) through glycolysis by transferring a phosphate from 1,3 diphosphoglycerate to adenosine diphosphate (ADP) in the sixth reaction of glycolysis [55]. Simultaneously, PGK1 can also stimulate pyruvate dehydrogenase kinase and inhibit the entrance of pyruvate into the mitochondrion (Krebs cycle), increasing lactic acid production in the cytosol which is then exported to other tissues (Figure 2) [56]. Interestingly, increased expression of PGK1 in colon cancer tissue has been associated with metastasis in a cohort of patients with colon cancer [57].

Additionally, even though overactivation of IL-6 has been extensively linked to muscle degradation in cancer cachexia, IL-6, released by contracting skeletal muscle, has been demonstrated to contribute positively to glucose muscle uptake and adipose tissue mobilization after a single bout of aerobic exercise in a model of IL-6 knockout mice [58]. This positive effect on skeletal muscle metabolism of muscle IL-6 is thought to generate a crosstalk between adipose tissue and skeletal muscle, and it can also explain the controversial inflammatory results found in some studies [59]. Overexpression of IL-15, another cytokine with anti-inflammatory properties, has been shown to attenuate muscle fatigue by improving oxidative capacity in skeletal muscle of a mouse model of breast cancer [60].

In humans, obese elderly patients have shown a reduction in IL-6 and TNF expression in skeletal muscle after 12 weeks of combined aerobic and resistance exercise, while a diet-induced weight loss intervention had no effect on inflammatory markers [61]. Moreover, whilst combined exercise training may have an impact on reducing inflammation, the absence of physical activity has been shown to increase muscle levels of IL-6 expression after a 7-day period of bed rest in older adults [62]. However, we still lack similar results in patients with cancer, especially in association with cancer cachexia.

TNF, initially called cachectin, combined with IL-6 induces activation of the nuclear factor kappa-light-chain-enhancer of activated B cells (NF-κB) [63]. NF-κB is an important regulator of genes related to tumorigenesis [64] and, in the skeletal muscle, may reduce protein into amino acids causing muscle atrophy, as shown in a cancer cachexia model [65]. Moreover, the ability of NF-κB to promote loss of muscle mass has been shown to be stimulated by chemotherapeutic agents [66,67] as well as muscle-specific E3 ubiquitin ligases intermediate of the UPS, such as muscle RING (really interesting new gene) Finger-1 (MuRF-1), and muscle atrophy F-box (MAFbx)/atrogin-1 (Figure 2) [65]. Although STAT3 and NF-κB may regulate genes expression in a cooperative manner, the role of NF-κB in muscle metabolism is still poorly understood [49].

It has been postulated that MuRF-1 may be responsible for degrading contractile and structural muscle proteins, such as titin, troponin-1, myosin heavy, and light chains, and proteins associated with glycolysis/glycogenolysis, whereas MAFbx/atrogin-1 is involved in impaired muscle protein synthesis, including MyoD as substrate [68,69]. These alterations in MuRF-1 and MAFbx/atrogin-1, however, seem to take place even before the presence of weight loss [70] or in early stages of cancer cachexia [71], whilst patients with cancer cachexia have been shown to present an inverse relation between the muscle expression of ubiquitin mRNA and the level of weight loss [72].

In addition, the mammalian target of rapamycin (mTOR), a serine/threonine protein kinase, is composed of two distinct complexes termed as mTOR complex 1 and 2 (mTORC1 and mTORC2, respectively) and is responsible for the main signaling pathway in cell growth and proliferation. It works through a cascade that starts with growth signals (e.g., insulin-like growth factor 1; IGF-1) phosphorylating the receptor tyrosine kinase, followed by activation of phosphoinositide 3-kinase (PI3K) and protein kinase B (Akt) (Figure 2) [73]. Interestingly, mTORC1 stimulates protein and lipid synthesis while suppressing catabolic pathways involved in autophagy [74]. However, IL-6 has been shown to suppress mTOR activity in a dose-dependent manner in human skeletal muscle and cultured C_2_C_12_ myotubes by activation of AMP-activated protein kinase (AMPK), but not Akt (Figure 2) [75].

With regards of the reduced activity of mTOR that may lead to impaired suppression of autophagy, several studies have demonstrated that intermediates of the autophagic-lysosomal proteolytic system are also increased in patients with different types of cancer [76,77,78]. Thus, these modifications in the suppression of mTOR associated with elevated autophagy further support the concept that autophagy may also be exacerbated in patients with cancer cachexia.

## 4. Insulin Resistance in Cancer Cachexia

The glucose uptake in muscle and adipose tissue is mediated by insulin that removes glucose from the circulation when there is an elevation in glucose concentration and simultaneously decreases glucose production in hepatic cells. However, unlike patients with type II diabetes mellitus characterized by chronic hyperglycemia, patients with cancer have normal level of fasting glucose, probably due to the redistribution of glucose to supply energy demand in cancer cells [79].

Chronic insulin resistance (IR) has been described in several types of cancer [80,81] and in early stages of cachexia in a mice model [82], but some studies have not shown any association between IR and loss of body weight in a cohort of unselected patients with cancer [83]. Of interest, insulin sensitivity has shown to be restored after surgical removal of the tumor, showing that the tumor may be the underlying cause of IR [84].

Recent genetic studies with phenotypes resembling cancer cachexia, using the fruit fly *Drosiphila melanogaster*, have found a tumor-secreted factor that was shown to be responsible for the wasting process in organs distant from the tumor [85]. This is an insulin growth factor binding protein (IGFBP) homolog called ImpL2 and its release may be involved in a crosstalk between tumor and muscle cell. Additionally, in another study with the same fly model, only malignant, but not benign, tumors promoted a downregulation of the insulin signaling pathway, which led to IR in peripheral tissues [86].

Furthermore, IR has been associated with reduced whole-body protein anabolism in male patients with NSCLC and this impaired anabolic response was mediated by increased proteolysis that increased amino acids in circulation (hyperaminoacidemia), leading to an exacerbated IR [81].

Concurrently, adipose tissue, as an endocrine organ, releases adiponectin that controls anti-inflammatory responses and regulates glucose and lipid metabolism [87]. Under healthy physiological condition, adiponectin inhibits IL-6 and TNF and seems to have a functional interplay with IGF-1 that may be impaired in cancer patients [88,89]. However, adiponectin has been shown to be reduced in patients with cancer cachexia and its combination with cytokines released by the tumor microenvironment may exacerbate the inflammatory response seen in cancer patients, which further may lead to IR without changes in plasma glucose concentration [79].

Clinical investigations on the interaction between adipocytes/cancer cell byproducts and IR, as well as growth factor, in the development and progression of cancer cachexia are still scarce and well conducted studies with tissue sampling along the cancer trajectory are needed.

## 5. Brown Adipose Tissue and Metabolism

Adipose tissue is a vital organ in controlling body composition through the regulation of energy homeostasis, though it is commonly overshadowed by the increasing attention given to muscles. In humans, two distinct types of adipose tissue have been described, white adipose tissue (WAT) and brown adipose tissue (BAT), they are responsible for energy storage and hypothermia, respectively. BAT maintains body temperature by heat generation that involves an increase in the adipose tissue expression of uncoupling protein 1 (UCP1) and also regulates glucose and lipid metabolism [90].

Classical brown adipocytes are derived from a myf-5 cellular lineage, while white adipocytes are derived from a non-myf-5 lineage [91]. The latter has been also termed beige or brite cells when stimulated via browning, a process that has been characterized by the gradual conversion of white adipocytes into brown like cells (especially in the abdominal region) during the progression of cancer cachexia [10]. NF-κB p65, a subunit of NF-κB, has been shown to be upregulated in WAT promoting inflammation in this tissue in cachectic patients [92].

In addition, this thermogenic effect of BAT has been suggested to enhance resting energy expenditure and lipid mobilization (Figure 2). Although the effect of browning has been described to be similar across metabolic disorders such as obesity, diabetes mellitus and cancer, these alterations in lipid metabolism and energy expenditure have proved to be detrimental in the context of cancer cachexia [10].

The modulation of BAT seems to depend on Prep1, a proposed regulator of adipo-osteogenesis, that has been shown to induce reductions in WAT volume associated with increase in BAT density and reduced osteogenesis in a mice model [93]. Moreover, the activation of BAT is mediated by β-3 adrenergic receptor that is activated by the sympathetic nervous system leading to fat cell shrinkage (Figure 2) [94].

IL-6 also plays an important role in mediating BAT activation throughout the course of cachexia (early to late-stage cancer cachexia) in gastric and colorectal patients [95], via increased UCP1 expression and genes related to the β-oxidation of fatty acids that activate thermogenesis as shown in a mice model of colorectal tumor [96]. Additionally, in a Lewis lung carcinoma model, it has been shown that tumor-derived parathyroid-hormone-related protein (PTHrP) regulates the gene expression involved in adipose tissue thermogenesis (Figure 2), lipolytic enzymes, and muscle atrophy (myostatin, MuRF-1, and MAFbx/atrogin-1), whereas blocking PTHrP, even in the presence of increased tumor size, promoted maintenance of muscle and fat mass in these mice [94].

Interestingly, in a cohort of 47 patients with NSCLC or colorectal cancer, 17 of these patients presented higher levels of PTHrP accompanied by lower LBM and increased resting energy expenditure [94]. Although these results suggest a browning/thermogenic effect of adipose tissue on wasting of either fat or muscle mass, more prospective studies need to be conducted to elucidate the real contribution of fat mass in inflammation and body composition changes in patients with cancer cachexia.

## 6. Role of Liver Cells in Metabolism

The liver is the main organ, along with muscle and adipose tissue, responsible for orchestrating the requirement and distribution of energy to support systemic metabolism. Recently, some studies have suggested that tumor can promote structural and metabolic changes in liver cells, which may contribute to greater inflammation and metabolic derangement in cancer cachexia (Figure 1) [97,98]. Paradoxically, protein synthesis in hepatocytes is increased while, as mentioned above, it is reduced in skeletal muscle [10].

The interaction between liver and cancer cells is thought to be mediated by mononuclear cells through IL-6 activation [99]. Moreover, in patients with pancreatic cancer and cachexia, the infiltration of macrophages into liver tissue has been shown to trigger liver parenchymal cells to induce the production of pro-inflammatory cytokines resembling IL-6 [100]. In addition, IL-4, a cytokine with anti-inflammatory properties, has been shown to be downregulated in hepatocytes of pancreatic patients with cancer cachexia [101]. These adaptations in liver cells may exacerbate the imbalance between pro- and anti-inflammatory responses of patients with cancer cachexia leading to the progression of the disease.

Furthermore, reduced ATP synthesis and elevated energy wasting have been reported in hepatocytes mitochondria of a mice model of peritoneal carcinosis. Cardiolipin, a protein essential to liver oxidative phosphorylation, has shown to be increased in the cachectic mice [102] and biosynthesis dysregulation of cardiolipin has been mediated by TNF in vitro [103]. In humans, steatosis in hepatocytes has been described in cachectic patients [104].

Of interest, L-carnitine, a transporter of fatty acids matrix into the of the mitochondrion, has prevented the progression of non-alcoholic steatohepatitis and increased the expression of genes related to mitochondrial β-oxidation, while suppressing oxidative stress in hepatocytes of a mice model [105].

Additionally, in a model of C26 adenocarcinoma mice, L-carnitine upregulated the expression of carnitine palmitoyltransferase I/II (CPT I and CPT II) in liver tissue and reduced the serum levels of IL-6 and TNF [106]. In a further study, L-carnitine has shown to decrease NF-κB p65 expression by suppressing peroxisome proliferator-activated receptor-gamma and alpha (PPAR-γ and α) in a CPT I-dependent manner, suggesting that L-carnitine may be involved in liver inflammation and to a lesser extent in the systemic inflammation [107]. Interestingly, L-carnitine has shown to increase BMI in patients with advanced pancreatic cancer with fat mass being a major contributor in this process [108].

PPAR-α suppression and increased gluconeogenesis in hepatic metabolism have been shown to be the cause of reduced serum ketones in a mouse model of NSCLC, whereas the restoration of ketone production in liver cells via PPAR-α agonist agent (fenofibrate) prevented weight loss and attenuated the degradation of type II muscle fibers compared to control fasted mouse [109]. Taken together, these findings involving PPAR-α and L-carnitine suggest that tackling hepatic metabolism can improve cancer cachexia status, although the mechanisms may be different between cancer types.

## 7. Treatment Perspective

Patients with cancer cachexia often experience reduced physical function, higher symptom burden, and poor quality of life (Figure 1) [110,111]. Cancer cachexia requires a multimodal approach in which drug therapies, nutritional support, and physical exercise must be included. However, an ideal pharmacological candidate to counteract cancer cachexia is still under development and should be an agent that targets systemic inflammation, improves body compartments while enhancing anabolism/catabolism balance, and in particular hinders anorexia [112,113].

Even though the pathogenesis of cancer cachexia is not fully understood, several candidates to prevent the loss of muscle and fat mass have been tested in experimental studies, including leucine and fish oil [114], rosiglitazone (insulin sensitizer) [115], activin receptor type 2 blockers [116], trimetazidine (exercise mimetic) [117], bortezomib (NF-κB inhibitor) [118], and vitamin D supplementation [119].

In addition, other agents have already reached clinical trials, such as myostatin inhibitors [120], the appetite stimulant megestrol acetate [121], testosterone [122], antimyostatin antibody [123], and monoclonal antibody (MABp1) [124], showing the influence of these drugs in different aspects of the spectrum of cancer cachexia. Recently, a combination of drugs to treat patients with cancer cachexia has been tested [125]. On the other hand, some compounds, including thalidomide, produced side effects that may outweigh their potential benefits [126].

Ghrelin, the so-called hunger hormone, has been demonstrated to produce an anti-inflammatory effect and inhibit pro-inflammatory cytokines in cachectic patients [127], while improving food intake, gastric motility, modulation of taste, and glucose metabolism [128]. Anamorelin, a selective ghrelin-receptor agonist, has been demonstrated to mimic the action of growth factors (e.g., IGF-1 and GH), showing its potential to treat patients with cancer cachexia. In a series of phase 3 clinical trials in patients with NSCLC (ROMANA 1, 2 and 3), anamorelin, compared with placebo, was shown to improve body weight and symptoms related to cancer cachexia in all studies, whereas LBM and fat mass were increased in ROMANA 1 and 2 lacking changes in muscle strength across all trials [129,130].

Another advanced studied compound for muscle wasting is the selective androgen receptor modulators (SARMs), an oral non-steroidal compound that resembles testosterone in its action with limited side effects. SARMs have successfully demonstrated significant improvement in LBM combined with increased time and power on the stair climb test in patients with cancer (NSCLC, colorectal cancer, non-Hodgkin lymphoma, chronic lymphocytic leukemia, and breast cancer), independent of dose administration [131].

Additionally, because patients with cancer often develop cardiovascular alterations due to chemotherapy agents, β-blockers have also been proposed to treat and prevent cardiac and muscle wasting in such patients [132,133]. Espindolol, classified as a non-selective β-blocker, seems to mitigate the effects of cancer cachexia in three potential therapeutic steps: (1) by reducing catabolism via β receptor blockade, (2) by decreasing fatigue and thermogenesis, and (3) by enhancing anabolism via agonist action on β2 receptor [134].

In a phase II clinical trial (ACT-ONE trial), patients with colorectal cancer and NSCLC (phase III and IV) were allocated to either placebo, low-dose (2.5 mg bd), or high-dose (10 mg bd) espindolol treatment for 16 weeks. High-dose group showed greater gain in LBM with maintenance of fat mass compared to placebo and further associated with handgrip strength, while there was not statistically differences between low- and high-dose, and low-dose and placebo for these parameters [134].

To date, anamorelin, SARMs, and espindolol are the most promising candidates proposed to treat patients with cancer cachexia, showing an important improvement in LBM with, still, controversial impact on clinical outcomes (handgrip strength and physical performance) across trials. Therefore, more randomized, double-blind, placebo-controlled studies must be conducted to test these agents.

An interesting approach to tackle cancer cachexia is a combination of treatment modalities, including exercise training, nutrition intervention, and pharmacological agent, which is currently tested in the MENAC trial [135]. The aim of this trial is to mitigate or reverse the effects of cachexia in patients with cancer. Considering a broader multimodal intervention, knowledge, awareness, and regonition of cancer cachexia must be implemented earlier in medical schools to prepare healthcare professional to treat patients with such conditions [136].

In fact, aerobic exercise training has been associated with positive healthy-related outcomes, such as reduced systemic inflammation, improved immune function, and enhanced metabolism, which may be replicated in patients with cancer [137]. Although the mechanisms behind these adaptations are not fully elucidated, it has been suggested that exercise may metabolically compete with tumors and cause a redistribution of energy substrates, which in turn deprive energy supply for tumor cells [138]. Moreover, resistance exercise training has shown to increase muscle mass and muscle strength in patients with prostate cancer [139], while muscle mass loss is attenuated with combined aerobic and resistance training compared to only aerobic exercise in obese elderly patients [140].

Another treatment modality that may improve cancer cachexia status is nutrition supplementation. Protein intake between 1.2–1.5 g/kg/day has been suggested to treat patients with cancer and low muscle mass, but the nutrition intervention may not be limited to protein intake alone and should include other amino acids and derivatives, such as leucine (2–4 g/day), glutamine (0.3 g/day), creatine (5 g/day), and carnitine (4–6 g/day) [141].

## 8. Conclusions

In summary, metabolic dysfunction related to cancer cachexia still remains a challenge. The pathophysiology of cachexia is multifactorial and mediated by an interplay between the release of cytokines from the tumor and some organ of the host, including liver, muscle, and adipose tissue. These cytokines produce an imbalance between pro- and anti-inflammatory responses that change glucose, lipids, and protein metabolism, restraining anabolism while a catabolic state is sustained.

Moreover, several drugs have been tested in experimental studies and others have already yielded positive results in phase 2 and 3 clinical trials, showing improvements in lean body mass, but not physical function. Nonetheless, along the years, targeting a single therapy has not led to effective treatment of this condition, so further studies must focus on an integrated approach, which includes pharmacological agents, nutritional support, and physical exercise, to better understand the complex interaction that culminate in the wasting of body compartments in patients with cancer cachexia.

## Abbreviations

ADP	Adenosine diphosphate
Akt	Protein kinase B
AMPK	AMP-activated protein kinase
ATP	Adenosine triphosphate
BAT	Brown adipose tissue
BMI	Body mass index
CPT I	Carnitine palmitoyltransferase I
CPT II	Carnitine palmitoyltransferase II
EGF	Epidermal growth factor
GH	Growth hormone
HSL	Hormone-sensitive lipase
IFN-γ	Interferon-gamma
IGFBP	Insulin growth factor binding protein
IGF-1	Insulin-like growth factor 1
IL-1β	Interleukin-1 beta
IL-2	Interleukin-2
IL-4	Interleukin-4
IL-6	Interleukin-6
IL-10	Interleukin-10
IR	Insulin resistance
JAKs	Janus kinases
LBM	Lean body mass
MABp1	Monoclonal antibody
MAFbx	Muscle atrophy F-box
mTOR	mammalian target of rapamycin
mTORC1	mTOR complex 1
mTORC2	mTOR complex 2
MuRF-1	Muscle RING Finger-1
NF-κB	Nuclear factor kappa-light-chain-enhancer of activated B cells
NSCLC	Non-small cell lung cancer
PGK 1	Phosphoglycerate kinase 1
PI3K	Phosphoinositide 3-kinase
PPAR-γ	Peroxisome proliferator-activated receptor-gamma
PPAR-α	Peroxisome proliferator-activated receptor-alpha
PTHrP	Tumor-derived parathyroid-hormone-related protein
SARMs	Selective androgen receptor modulators
STAT3	Activating the signal transducer and activator of transcription 3
TNF	Tumor necrosis factor
UCP1	Uncoupling protein 1
UPS	Ubiquitin-proteasome system
WAT	White adipose tissue
